# Defect Engineering in 2D Materials: Precise Manipulation and Improved Functionalities

**DOI:** 10.34133/2019/4641739

**Published:** 2019-12-02

**Authors:** Jie Jiang, Tao Xu, Junpeng Lu, Litao Sun, Zhenhua Ni

**Affiliations:** ^1^School of Physics, Southeast University, Nanjing 211189, China; ^2^SEU-FEI Nano-Pico Center, Key Laboratory of MEMS of Ministry of Education, Southeast University, Nanjing 210096, China

## Abstract

Two-dimensional (2D) materials have attracted increasing interests in the last decade. The ultrathin feature of 2D materials makes them promising building blocks for next-generation electronic and optoelectronic devices. With reducing dimensionality from 3D to 2D, the inevitable defects will play more important roles in determining the properties of materials. In order to maximize the functionality of 2D materials, deep understanding and precise manipulation of the defects are indispensable. In the recent years, increasing research efforts have been made on the observation, understanding, manipulation, and control of defects in 2D materials. Here, we summarize the recent research progress of defect engineering on 2D materials. The defect engineering triggered by electron beam (e-beam), plasma, chemical treatment, and so forth is comprehensively reviewed. Firstly, e-beam irradiation-induced defect evolution, structural transformation, and novel structure fabrication are introduced. With the assistance of a high-resolution electron microscope, the dynamics of defect engineering can be visualized *in situ*. Subsequently, defect engineering employed to improve the performance of 2D devices by means of other methods of plasma, chemical, and ozone treatments is reviewed. At last, the challenges and opportunities of defect engineering on promoting the development of 2D materials are discussed. Through this review, we aim to build a correlation between defects and properties of 2D materials to support the design and optimization of high-performance electronic and optoelectronic devices.

## 1. Introduction

Two-dimensional (2D) materials with ultrathin thickness have shown extraordinary optical, electronic, and optoelectronic properties and allow higher integration density compared to traditional 3D semiconductors [1]. These stimulate the research of 2D materials on next-generation electronics and optoelectronics, including the transistor [2], photodetector [3], modulator [4], and light-emitting diode (LED) [5]. However, the properties of 2D materials and performances of devices built on 2D materials are generally inferior to theoretical prediction. The discrepancies between theory and reality mainly arise from the inevitable intrinsic defects in 2D materials.

Common defects such as vacancy, antisite, substitution, adatom, and grain boundary have been observed in 2D materials [6–9]. They have great influence on the optical, electronic, and optoelectronic properties of 2D materials [10–17]. Defects could act as carrier donors [14], scattering [11], trap [15], and recombination [16] centers in different conditions. For example, the sulfur vacancies (SVs) in MoS_2_ induce electron donor states in the electronic bandgap, which increases the electron concentration and induces n-type doping [13, 14]. As a result, the excess electrons trend to form negative trions instead of neutral excitons and lead to low photoluminescence (PL) quantum yield (QY) [13]. In addition, the defects including vacancies, charged impurities, and grain boundaries in 2D materials can act as scattering centers, increasing the carrier scattering probability and resulting in low carrier mobility [12, 14]. The defect-induced trap states will also prolong the response time of 2D photodetectors [15].

On the other hand, the properties of 2D materials can be modulated by defect engineering [18, 19]. Eliminating unfavorable defects and introducing beneficial defects are the strategy of defect engineering to realize higher performance electronic and optoelectronic devices. For example, SVs which have great impact on the properties of 2D materials can be passivated by O substitutions through oxygen plasma to enhance the PL QY [13], healed by thiol chemistry to improve the mobility [20], or partly passivated by molecules to reduce the response time while maintaining high responsivity [21]. Numerous techniques including electron beams (e-beams), plasma, chemical treatment, ozone, and laser have been developed to trigger defect engineering in 2D materials.

In this article, we provide a review on the recent progress of defect engineering in 2D materials triggered by e-beams, plasma, chemistry, ozone, and so forth. Firstly, we focus on the defect engineering triggered by the e-beam. With the assistance of high-resolution electron microscope (EM), e-beams can not only precisely modify the defects in 2D materials with atomic resolution but also visualize the dynamics of defect engineering *in situ*. The functionalities including defect generation and manipulation, structural transformation and phase transitions, and novel structure fabrication realized by e-beam irradiations are described successively. Subsequently, other methods such as plasma, chemical, and ozone treatments employed to trigger defect engineering in 2D materials are reviewed. The plasma treatment-induced defect engineering on the improvement of PL QY and the influence of metal contact and carrier mobility in 2D materials are introduced. The healing and doping effects of defects induced by chemical treatments are then discussed. Afterwards, we emphasize the defect engineering triggered by ozone treatment, laser illumination, substitutional doping, and ion implantation. At last, we provide our own view on the challenges and opportunities of defect engineering on promoting the development of 2D materials. The purpose of this review is to make a correlation between the concentrations and types of defects in 2D materials and their optical, electronic, and optoelectronic properties and support the design and optimization of high-performance electronic and optoelectronic devices by means of defect engineering.

## 2. Engineering 2D Materials by e-Beam Irradiation at the Nanoscale

e-beam irradiation is usually considered to introduce disorders into materials and spoil their properties, which is undesirable and often referred to as damage. However, it may have a beneficial effect on nanomaterials especially when combined with heat treatment. A large number of experimental studies show that the atomic structure and morphology can be modified in a controllable manner by e-beam irradiation [22–25], which makes e-beam irradiation an efficient technique to modify the properties of nanomaterials.

When an energetic electron penetrates 2D sheets, it collides with the nuclei and the electrons surround the nuclei in the specimen. Only a small amount of energy can be transferred from the incident electron to the nucleus via electron-nucleus collision upon energy and momentum conservation, so that very high electron energy (threshold energy *E*^*th*^, normally larger than tens of keV, Table 1) is needed to knock an atom deviating from its lattice. This process is usually referred to as knock-on damage or displacement, and the displacement rate of each atom is proportional to the beam current density. Electron-electron collision, on the other hand, is able to stimulate ionization or bond breaking even at the energies much lower than the knock-on damage threshold, which does not lead to atom displacement but may damage the specimen via local chemical reaction. Hence, both electron-nucleus collision and electron-electron collision can tailor local structures in predetermined areas of the specimen by controlling the e-beam (including energy, dose, and irradiation area). Furthermore, activating phase transitions and sculpting novel nanostructures are feasible via controlled e-beam irradiation. This demonstrates the ability of the e-beam to fabricate building blocks for nanodevices.

In this section, we focus on e-beam-assisted defect evolution, structural transformation, and novel structure fabrication in EM, which can not only trigger structural changes but also visualize the dynamic processes *in situ*. In particular, the state-of-the-art transmission electron microscope (TEM) equipped with an aberration corrector is able to monitor structural evolution of the 2D monolayer at the atomic scale. The full microscopic pictures may promote the understanding of e-beam irradiation-driven processes, which represents a significant step forward to engineering 2D materials with atomic precision.

### 2.1. Generation and Manipulation of Defects

**Figure 1:**
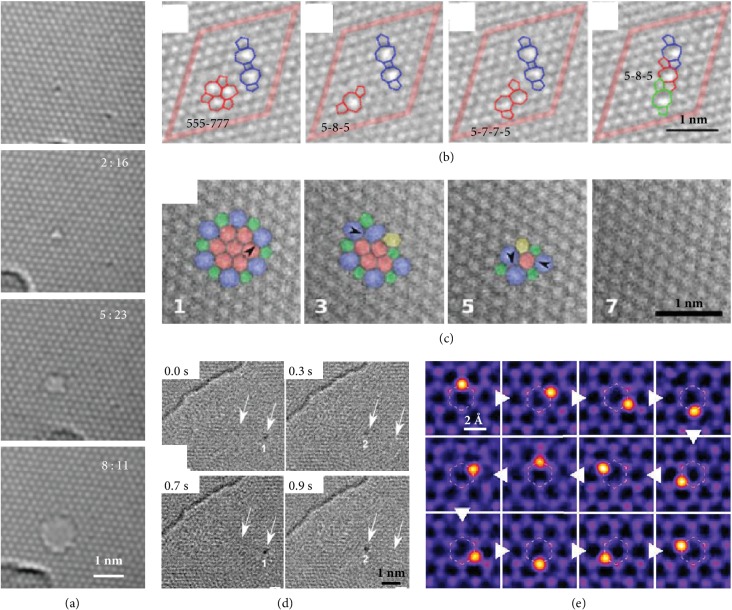
Generation and manipulation of defects in 2D materials by e-beam. (a) Creation of vacancies in graphene under 80 keV e-beam irradiation. (b) Electron beam-driven divacancy migration observed at 80 keV. Reproduced from [30]. (c) Configurational changes of flower defect via C-C bond rotations in graphene. Reproduced from [31]. (d) Oscillations of a W atom between two trapping centers 1 and 2 on a few-layer graphene at 480°C. Reproduced from [33]. (e) e-beam manipulation of a Si atom around a single hexagon in graphene. Reproduced from [37].

2D materials are sensitive to e-beam irradiation, and knock-on damage is the most relevant process on 2D materials [23]. The defects can be easily created when the incident energy is larger than the knock-on damage threshold. In the case of graphene, the knock-on damage occurs at electron acceleration voltages above 80 kV at room temperature, which means that pristine graphene is stable at 80 kV. However, the vacancy can be introduced at 80 kV if there are surface absorbates (Figure 1(a)), which is likely due to the reaction between the adsorbates and graphene with the assistance of e-beam irradiation. Similar phenomena are also frequently observed in semiconducting transition metal dichalcogenides (TMDCs). The anion vacancy can be generated in monolayer TMDCs under the e-beam at a voltage much lower than the knock-on damage threshold, which probably proceeds via ionization damage or is catalyzed by surface contaminants [26].

It is a remarkable fact that the knock-on damage threshold is dependent on chemical composition and atomic arrangement. Take *h*-BN as an example; the threshold for B (74 kV) is lower than N (84 kV), and thus, B vacancies are frequently created in *h*-BN sheets irradiated by an 80 kV e-beam [27]. On the other hand, the threshold declines sharply in the presence of atoms with unsaturated bonds, and thus, the atoms in these regions can be sputtered off easily. As a consequence, the single vacancy in Figure 1(a) can evolve into a circular hole with high symmetry at 80 kV. It should be noted that the shape of the as-created hole is dependent on the crystal structure of the 2D sheet; for example, triangular-shaped holes with N-terminated zigzag edges are frequently created in the monolayer *h*-BN at room temperature by e-beam irradiation [27], while parallelogramic-shaped and hexagonal-shaped holes with both B- and N-terminated edges become prominent at temperatures above 700°C [28]. The geometry and edge configurations have significant influence on the performance of a nanohole-based device [29].

Point defects can also be created via bond rotations and keep the atom-number constant under the e-beam irradiation. In *sp*^2^-bonded carbon structures, the activation energies for bond rotation are much lower than those for carbon displacements. Correspondingly, C-C bond rotations can take place in pristine graphene at 80 kV, resulting in the formation of the Stone-Wales defects or structural transformation between different configurations. As shown in Figure 1(b), the 5-8-5, 555-777, and 5-7-7-5 divacancies in graphene can convert to each other under e-beam irradiation [30]. Another typical example is the formation and annihilation of the close-loop “flower defect” in pristine graphene [31]. The flowerlike structure with a core of seven hexagons rotated by 30° can be created via six consecutive bond rotations. Conversely, such a structural defect without atom loss can relax to a less disordered state or even turn back to the pristine lattice by C-C bond rotation, as shown in Figure 1(c).

Further e-beam bombardment can trigger the migration, agglomeration, and reconstruction of point defects if the transfer energy is comparable to the atom binding energy, which provides a way to manipulate the structures at the nanoscale and even atomic scale. For graphene, by carefully choosing the electron energy, e-beam-driven vacancy agglomeration can lead to the formation of multivacancy structures constructed of rotated hexagons and other polygons [30]; the vacancies can also reorganize into a missing row of atoms in between the two dislocation cores [32]. The dislocation core consisting of a pentagon-heptagon pair can migrate by both bond rotation-mediated gliding and atom removal-induced climbing, which can be activated by the e-beam [32].

Foreign atoms can be trapped by point defects in 2D sheets due to the attractive interaction resulted from the local strain field. e-beam-driven manipulation can occur at these impurity sites. As shown in Figure 1(d), an adsorbed W atom can jump forth and back repeatedly between two adjacent divacancies in graphene with a distance of 0.5-1.4 nm under e-beam [33]. Other impurity atoms in graphene also present similar behaviors under e-beam irradiation, such as the oscillation of pyridinic-N substitution between equivalent bonding sites across a monovacancy [34] and the movement of Si substitution via out-of-plane bond inversion [35]. These movements can be well controlled by purposefully directing the electron irradiation at the desired position inside a scanning TEM where the e-beam can be focused onto the atomic scale. Recent experiments have demonstrated that the movement of Si impurities within graphene sheets can be controlled by parking the e-beam for seconds on top of the C neighbor in the direction the Si should move [36–38], and then the Si can be precisely moved along an extended path, such as circulating a single hexagon in Figure 1(e).

### 2.2. Structural Transformation

Structural transformation can be achieved by knock-on collisions, radiolysis, or charging or by other mechanisms. Hence, complete phase transitions of 2D materials is feasible via controlled e-beam irradiation.

One example is the transformation from crystalline to an amorphous 2D membrane of *sp*^2^-hybridized carbon atoms when the graphene sheet is exposed to the e-beam with energy just above the threshold of the knock-on damage [30]. e-beam irradiation produces defects of 30° rotated hexagons surrounded by alternating pentagons and heptagons, which are the energetically favored way for the graphene lattice to accommodate the C loss. The expansion of these defects results in a transition from a crystalline structure to a state approaching a random network. Conversely, hydrocarbon adsorbates on 2D sheets can transform into amorphous C layers [39] and then transform into graphene sheets parallel to the underlying substrate [40]. Such a transformation is attributed to e-beam-induced heating and the van der Waals interaction from the substrate. Similarly, amorphous MoS_2_ deposited on the graphene substrate can restructure into crystalline domains under e-beam irradiation [41].

Normally, substitutions are energetically favored with respect to the isolated atoms; hence, substitution doping can be achieved by healing of vacancies with foreign atoms if both 2D materials and the feedstock for substitutions are exposed to electron bombardment. For instance, B and N vacancies generated by electron irradiation will be filled by C atoms when a BN sheet loaded with paraffin wax as a carbon source is exposed to the e-beam, resulting in the transformation from the insulating BN sheet into conducting BCN sheets [42]. BN honeycomb lattices can nearly completely be substituted by C atoms, demonstrating that e-beam-induced doping can tune the electrical properties of BCN structures in a full range of ternary BCN compositions.

e-beam irradiation also affects the functional properties of 2D materials. For example, insulating fluorinated graphene can be reduced by e-beam irradiation and then transformed into a conducting or semiconducting structure [43]. As reported by Withers et al., e-beam irradiation monotonously decreases the resistivity of fluorinated graphene, up to 7 orders of magnitude; the resistance of the fluorinated graphene decreases with increasing channel width (*W*) following a 1/*W* dependence. These findings demonstrate that e-beam patterning opens up new ways for the fabrication of all-graphene electronics where fluorinated graphene is used as the insulating host and defluorinated graphene is used as the metallic interconnect or active device element; patterning channels with different conductivities also have potential applications in resistive memory and data storage [43].

**Figure 2:**
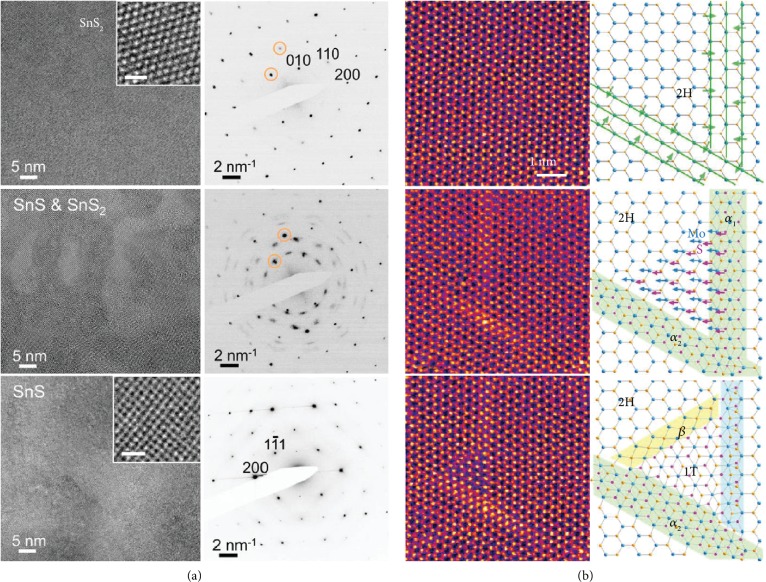
Phase transformation under electron irradiation. (a) The transformation from few-layer SnS_2_ to SnS. Reproduced from [44]. (b) Structural transformation between 2H and 1T in MoS_2_ via an intermediate *α*-phase. Reproduced from [45].

Electron irradiation can also drive the transformation between different structures if the layered materials have multiple stable phases with different stoichiometries. As shown in Figure 2(a), when tin dichalcogenide sheets are exposed to the e-beam at both room and elevated temperatures, the transformations driven by a progressive chalcogen loss initially result in mixed mono and dichalcogenides, followed by the complete conversion to highly anisotropic orthorhombic monochalcogenides [44]. It demonstrates the capability to tune the properties of layered crystals that have stable polymorphs with different stoichiometries.

Most of the above transformations are attributed to the loss of atoms. e-beam irradiation can drive the conversion between phases without a net loss of atoms. Take MoS_2_ as an example; e-beam irradiation can trigger the transformation between semiconducting 2H and metallic 1T phases, which involves lattice-plane gliding [45]. As shown in Figure 2(b), the 2H to 1T transition is initiated by the formation of an *α*-phase precursor with three to four constricted zigzag chains. When two nonparallel *α*-phases are in contact, the local strain triggers S-plane or Mo-S atom to glide to form a triangular nucleus of the 1T phase, which further expands via migration of the secondary boundary at the edge of the 1T phase. The phase transformation occurs only in the irradiation regions which can be controlled easily in a scanning TEM. Because the 1T and 2H phases have distinct electronic properties, this controllable local phase transition may enable bottom-up fabrication of nanoelectronics.

### 2.3. Fabrication of Novel Structures

Apart from modification of the atomic structure and properties of 2D materials, the e-beam can also be used to fabricate devices because the irradiation region can be easily controlled. Rodríguez-Manzo et al. have successfully fabricated a three-electrode device from a continuous graphene sheet where the third electrode operates as a side gate in a field-effect transistor (FET) [46]. The sculpted graphene nanoribbon was suspended between the source and drain electrodes and served as a FET channel where the carrier density can be modulated by the side-gate potential. Although the e-beam provides the possibility, *in situ* EM fabrication of nanodevices remains challenging due to the lack of operational flexibility.

Relatively, creation of novel structures with atomic precision as building blocks for devices is also very attractive. A typical example is the subnanometer quasi-1D structure, which is one of the most promising building blocks for future electronic devices. Direct e-beam irradiation of 2D materials provides a top-down strategy to fabricate such an ultrathin structure by controlling irradiation regions and the electron dose. Firstly, quasi-1D ribbons can be constructed between two adjacent pores, and the size of as-constructed ribbons strongly relies on the irradiation regions. The ribbons are further shrunk under prolonged e-beam irradiation, leading to the formation of extremely narrow structures and even atomic chains eventually. Atomic chains are first created in graphene via sputtering carbon atoms [47], and the as-formed carbon atomic chains show high flexibility. Experimental measurements confirm that the conductivity of the carbon chain depends on the local strain [48]. The current-voltage curve of an unstrained chain shows a linear behavior, which is in perfect accordance with the metallic cumulene with identical-length double bonds. If the chain is under strain, the S-shape current-voltage curve is shown, which demonstrates that the 1D system is a semiconducting polyyne chain with alternating single and triple bonds. The arrangement of carbon in chains might also be affected by temperature [49]. Two distinct arrangements coexist at low or ambient temperatures, while an unexpectedly high polyyne ratio is observed in carbon chains fabricated at elevated temperatures. Such a top-down method can be extended to other 2D systems. Atomic chains with alternating B and N atoms have been created in *h*-BN sheets under electron irradiation and they are expected to remain insulating [50]; phosphorus chains with zigzag configurations have also been created in phosphorene, and their stability is enhanced if the chains are supported by a substrate sheet [51].

**Figure 3:**
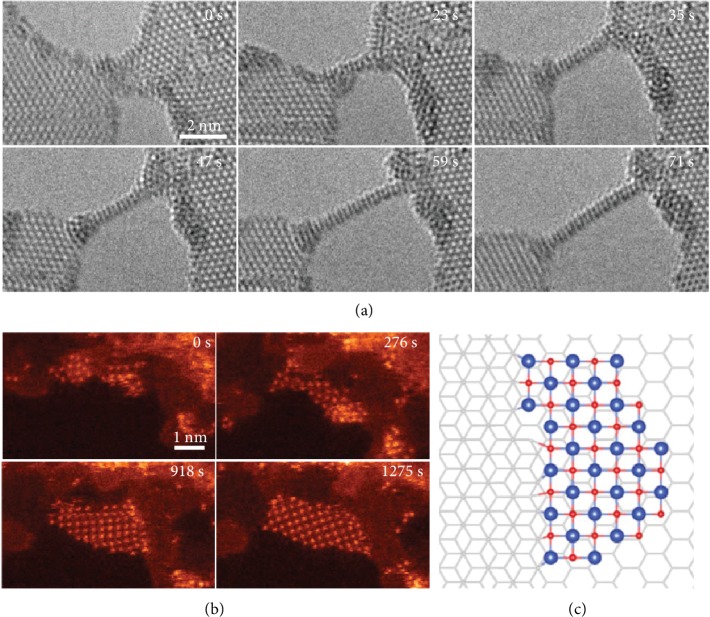
Novel nanostructures created in 2D materials under electron irradiation. (a) Subnanometer ribbon derived in MoS_2_ sheets under e-beam (80 kV, 10 A/cm^2^). Reproduced from [52]. (b, c) CuO monolayer grown on graphene substrate and corresponding theoretical model. Reproduced from [56].

Similarly, ultrathin wires can be constructed from semiconducting TMDCs [52–54]. As shown in Figure 3(a), ultranarrow wires can be derived from the monolayer MoS_2_ by further narrowing the ribbons between two adjacent pores [52]. These wires are robust under e-beam irradiation, and their atomic structures are obviously different from the initial MoS_2_. *In situ* electrical measurements show significant increase in conductance as the nanowire forms [53], which is a direct evidence of the conversion of the semiconducting monolayer to a metallic nanowire. On this basis, complex junctions and alloyed nanowires such as MoS_*x*_Se_1-__*x*_ can be created. It paves a way for robust ultrathin building blocks for future flexible electronics [54]. Single-walled tubular structures can also be created in bilayer sheets when the dangling bonds at ribbon edges are saturated with interlayer bonds [27, 55]. The electronic properties can be well modified by controlling the irradiation position [55].

With activation energy transferred from the e-beam, small clusters on 2D sheets can be assembled into crystalline monolayers, providing a way to synthesize novel quasi-2D materials. Single-atom-thick CuO layers have been created from CuO_*x*_ clusters both on graphene substrates (Figure 3(b)) and in graphene nanopores. The schematic diagram of the structure is shown in Figure 3(c) [56]. First-principle calculation suggests that monolayer CuO with a D-type antiferromagnetic ordering is an indirect wide-bandgap semiconductor. If one-half of O atoms are further removed by electron beam irradiation, monolayer CuO may transform into another stable oxide (Cu_2_O) with direct bandgap. The indirect-to-direct bandgap transition holds promise in applications in optoelectronics where the optical properties can be tuned by modulating the oxygen content.

## 3. Improving the Properties of 2D Materials by Defect Engineering

e-beams in EM have the capability to visualize the dynamics of structural changes *in situ* even in the atomic scale and promote the understanding of microscopic prospect in defect engineering. However, e-beam irradiations have limitations (e.g., operation area, cost, and efficiency) in modulating the properties of 2D materials to achieve high-performance devices. In the following parts, effective methods of defect engineering used to improve the properties of 2D materials will be introduced.

Plasma and chemical treatments are widely used in triggering defect engineering in 2D materials [18]. Plasma is the general denotation of a statistical system containing mobile charged particles. The energetic ions can react with matters and change their structures. The ions with kinetic energy could strike with the atoms to create vacancies and structural deformation. They will also react with the materials at the defect sites to form substitutional impurities and adatoms. The plasma irradiation can be easily controlled by plasma parameters (pressure, power, and time) and are immune from contamination due to the dry atmosphere. As another common method, chemical treatments can modulate the properties via reactions and charge transfer between 2D materials and chemicals. They are employed to heal the defects without the introduction of new defects by carefully selecting reactants [20, 57]. Meanwhile, they can also facilitate carrier transport [58], induce effective doping [59], and modulate the band and phase of structures [60]. Apart from plasma and chemical treatments, other techniques such as laser modification and ozone reaction are also utilized in defect engineering. They greatly enrich the means for studying and improving the properties of 2D materials.

In this section, we will describe the modulation of optical, electronic, and optoelectronic properties of 2D materials via macroscopic methods such as plasma and chemical treatment. Through defect engineering, high-performance electronic and optoelectronic devices based on 2D materials are achieved.

### 3.1. Defect Engineering by Plasma Irradiation

**Figure 4:**
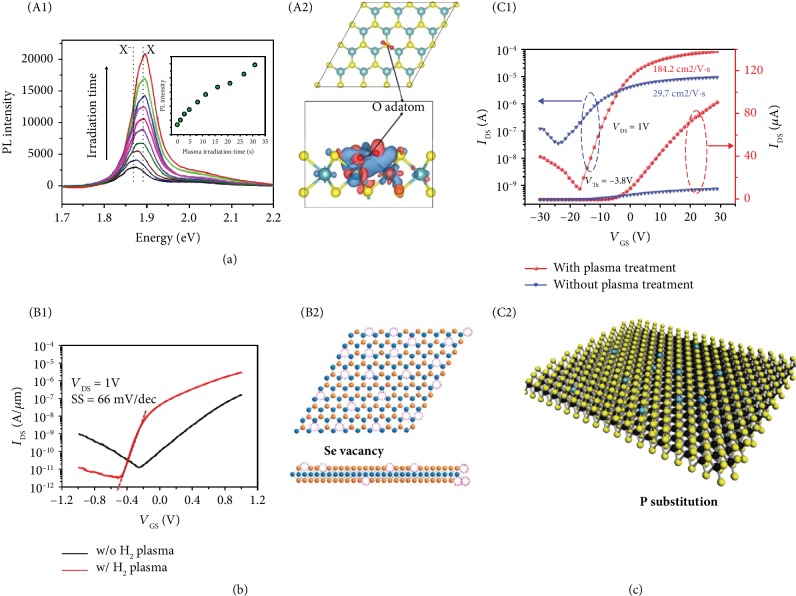
Defect engineering by plasma irradiation. (a) PL spectra of monolayer MoS_2_ after oxygen plasma irradiation with different durations (1) and relaxed configuration and charge density of an O_2_ molecule chemisorbed on a monosulfur vacancy of MoS_2_ (2). The positive and negative charges are shown in red and blue, respectively. Reproduced from [13]. (b) Transfer characteristic of WSe_2_ FET with/without H_2_ plasma treatment (1) and the schematic diagram of Se vacancies in WSe_2_ (2). Reproduced from [66]. (c) Transfer characteristic of WS_2_ with/without N_2_ plasma treatment (1) and the schematic diagram of P substitutions in WS_2_ (2). Reproduced from [75].

We firstly use MoS_2_ as an example to illustrate the defect engineering for modulating the optical properties by plasma treatment. The S vacancies in the MoS_2_ monolayer facilitate n-type doping and promote the formation of trions. This results in relatively low PL intensity in the MoS_2_ monolayer as excitons are radiative, whereas the dominant trion recombination pathway is nonradiative [61]. A strong PL enhancement of the monolayer MoS_2_ can be realized by mild oxygen plasma irradiation [13]. Oxygen adatoms on sulfur defects induce strong charge transfer (0.997 e, from MoS_2_ to O_2_), as illustrated by the charge density difference shown in Figure 4(a), leading to heavy p-doping and converting trions to excitons. Secondly, excitons localized at the defect sites generally have much larger binding energy, which suppresses the thermally activated nonradiative recombination and results in high PL QY. By careful control of experimental conditions, the PL enhancement could be as high as 100 times (Figure 4(a)). It should be noted that the parameters of plasma treatment are very important. PL quenching might be observed if the irradiation is too strong [62]. The PL intensity of the multilayer MoS_2_ can also be enhanced by oxygen plasma treatment [63]. The thickness of the multilayer MoS_2_ increases slightly after plasma irradiation because oxygen plasma insert into the interlayer of MoS_2_ and break the interlayer Van der Waals (vdW) bonding. This leads to indirect-to-direct bandgap transition, and it is in accordance with the change of calculated electronic band structures of the multilayer MoS_2_ with different interlayer vdW distances [63]. In addition, an enhancement of circular polarization in the PL emission spectrum is also observed in few layered MoS_2_ treated by remote oxygen plasma, which provides a solution to fabricate efficient spin polarized optoelectronic devices based on TMDC multilayers [64].

The electronic properties of 2D materials can also be tuned by plasma treatment, e.g., lowering the contact resistance, tailoring the n/p or ambipolar behaviors, and improving the carrier mobility. The contact resistance arises from the Schottky barriers (SBs) at the metal/semiconductor contact and it can be reduced by doping [65]. The Se vacancies introduced by H_2_/He plasma in WSe_2_ induce n-type doping, and the increased doping concentration would decrease the SB width [66]. As a result, more electrons can be injected into WSe_2_ by tunneling through the SBs and hence lowering the contact resistance by 2 orders of magnitude. It increases the ON current 20 times and shows a nearly ideal subthreshold swing value of 66 mV/dec (Figure 4(b)). Similarly, the formation of WO_3_ in WSe_2_ after N_2_O plasma treatment induces p-doping and reduces the contact resistance by 5 orders of magnitude [67]. The contact resistance can also be reduced by phase transition from the semiconducting to the metallic phase. Zhu et al. reported a 2H-1T phase transition in the monolayer MoS_2_ via weak Ar plasma bombardment [68]. The Ar ions with kinetic energies lead to the lateral sliding of the top S-layer of the 2H phase MoS_2_ and form the 1T phase structure. The power of plasma is well controlled to avoid the etching effect and formation of defects like vacancies. The phase transition is confirmed by scanning tunneling microscopy (STM) and scanning tunneling spectroscopy (STS). Metallic 1T phase MoS_2_ within the metal contact area reduces both SB height and width and hence lowers the contact resistance. The fabricated FET shows higher on/off ratio and larger ON current.

The n, p, or ambipolar behaviors are also dominated by SBs between metals and channel materials. The substitutional O atoms on the surface of MoS_2_ after oxygen plasma treatment induces p-doping and reduces the SB heights for holes [69]. As a result, partly oxidized MoS_2_ tunes the SB heights for electrons from a narrow distribution (from 0.2 to 0.3 eV) to a broader distribution (from 0.2 to 0.8 eV), which allows both electron and hole injections. By deposition of metal at the plasma-treated area, multilayer MoS_2_ FETs exhibit ambipolar current transport with a field-effect mobility of 11.5 and 7.2 cm^2^ V^−1^ s^−1^ for electrons and holes, respectively. PH_3_ plasma can also induce p-doping of MoS_2_ [70]. The fabricated MoS_2_ FET shows ambipolar behavior but with a dominated p-branch due to more uniform p-doping from substitutional P atoms. Flexible conversion of the n- and p-types in MoS_2_ makes it proper to fabricate high-performance complementary logic applications. A logic circuit built on a lateral homogenous p-n junction is hence realized in MoS_2_ to present high rectification ratio of 2 × 10^4^. N_2_ plasma not only induce p-doping in MoS_2_ but also bring forth compressive strain via nitrogen substitutions [71]. Such strain in self-assembled systems made of high critical temperature (*T*_c_) superconducting films containing nanocolumns of BaZrO_3_ will induce oxygen deficiency, leading to a significant reduction in *T*_c_ [72]. In addition, the superconducting properties of such films can be improved by strain-tuning [73].

Plasma has also been employed to improve the carrier mobility of 2D materials. Vacancies in 2D materials often serve as scattering centers and lead to lower mobility as compared to theoretical prediction. For example, S vacancies in MoS_2_ would generate localized states in the bandgap, resulting in hopping transport behavior and low mobility [14]. A WSe_2_ FET fabricated by e-beam lithography free process shows high hole mobility of 200 cm^2^ V^−1^ s^−1^ because of the absence of e-beam-induced vacancy defects [12]. Nan et al. demonstrated that S vacancies can be “repaired” by oxygen plasma treatment [74]. The bonding exciton peak induced by S vacancies almost vanishes after plasma exposure, which means most of the vacancies are filled by substitutional oxygen and the vacancy scattering centers are removed. As a result, the mobility of electrons in MoS_2_ is increased by about 48 times. Similarly, the sulfur vacancies of WS_2_ can be filled by nitrogen atoms after N_2_ plasma treatment, which is confirmed by STEM [75]. The density of SVs is reduced, leading to an improved mobility to 184.2 cm^2^ V^−1^ s^−1^ (Figure 4(c)).

### 3.2. Defect Engineering by Chemical Treatments

Chemical treatments are widely used in surface modifications. Compared to plasma irradiation, chemical treatments could provide efficient doping with less damage to the materials. Chemical treatments are also employed to modulate the trap states in optoelectronic devices.

**Figure 5:**
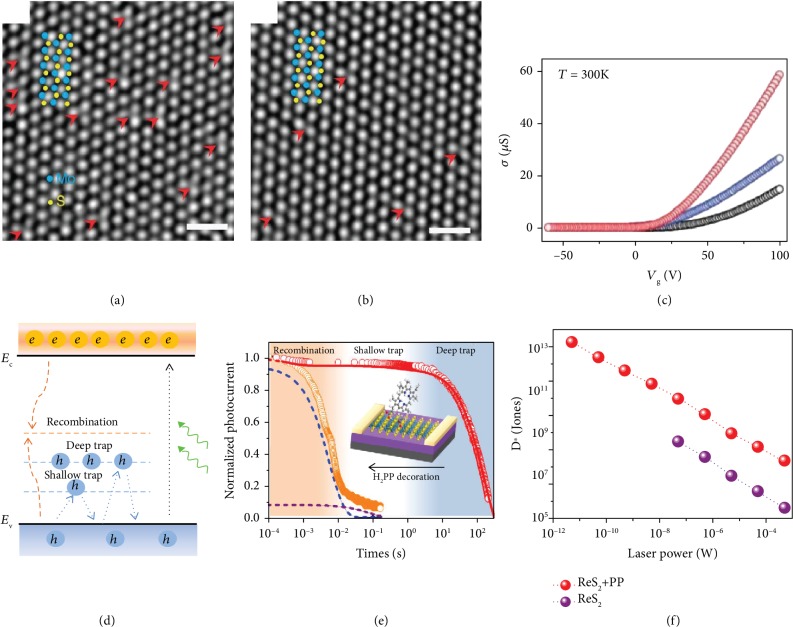
Defect engineering by chemical treatment. TEM image of MoS_2_ before (a) and after (b) MPS treatment. Scale bar, 1 nm. (c) Typical *σ*-*V*_g_ characteristics for as-exfoliated (black), TS-treated (blue), and DS-treated monolayer MoS_2_ at *T* = 300 K. Reproduced from [20]. (d) Schematic diagram of carrier recombination and trapping kinetics. (e) Transient response and (f) specific detectivity of as-prepared and H_2_PP-decorated ReS_2_. Reproduced from [21].

Thiol chemistry has been employed to heal the S vacancies in MoS_2_ [20]. The healing mechanism is dominated by chemical reaction. The reaction kinetics of S vacancies and trimethoxysilane (MPS) comprises two steps with an energy barrier of 0.51 eV and 0.22 eV, respectively. The low energy barrier can be overcome by low temperature annealing. After MPS treatment, the density of S vacancies is reduced by 4-folds, as indicated by statistical analysis of the S vacancy density in TEM images (Figures 5(a) and 5(b)). As a result, high mobility of >80 cm^2^ V^−1^ s^−1^ at room temperature is achieved in the monolayer MoS_2_ FET. It is much higher than the as-prepared sample (Figure 5(c)). Compared with oxygen plasma treatment, thiol chemistry has negligible capability to introduce new S vacancies into MoS_2_. S vacancy self-healing (SVSH) using poly(4-styrenesulfonate) (PSS) treatment is another method to heal the defects [57]. PSS plays the role of catalyst in the reaction. The PSS healing process can be described as that sulfur adatom clusters on the as-grown MoS_2_ are guided by the hydrogenation of PSS to fill the vacancies. Scanning TEM images of the as-grown MoS_2_ show that the sulfur vacancies (1S) and sulfur adatom clusters are absent in SVSH MoS_2_. The electron concentration of healed MoS_2_ is hence decreased by 643 times and a lateral homojunction with a perfect rectifying behavior is fabricated. The performance of homojunction is largely enhanced because the lattice defect-induced local fields are eliminated.

When referring to photodetectors, defects often play an important role in determining the performance. For a photoconductor, the photocurrent is proportional to the photoconductivity gain (G), which is proportional to carrier lifetime and mobility. The schematic diagram of carrier recombination and trapping kinetics is shown in Figure 5(d) [21]. Defect-induced trap states will trap photoexcited minority carriers and prolong the lifetime of majority carriers, leading to high photocurrent. On the other hand, the response time will be prolonged by the thermal reexcitation of trapped carriers into the conduction or valence band, which could be in the timescale of second or minute for some deep traps [21]. The intrinsic ReS_2_ photoconductor shows an ultrahigh responsivity of 88600 A/W with a response time of tens of seconds, which indicates the existence of abundant deep trap states induced by sulfur vacancies [15]. Protoporphyrin (H_2_PP) molecules are employed to improve the response speed of ReS_2_ through the modulation of trap states. The transient responses of as-prepared and H_2_PP-decorated ReS_2_ devices are shown in Figure 5(e). H_2_PP molecules could fill the S vacancies by chemical bonding. After the removal of most S vacancies, the shallow traps induced by the rest of the S vacancies start to dominate the response time of the device. As a result, decay time is shortened by several orders of magnitude. Moreover, the specific detectivity of the phototransistor is greatly enhanced due to the reduction of dark current originating from the charge transfer between ReS_2_ and molecules (Figure 5(f)).

Various chemical dopants have been utilized in doping of 2D materials. Modulation of PL could be realized by chemical doping. Amani et al. demonstrated near-unity PL QY in MoS_2_ and WS_2_ monolayers after being treated by an organic superacid: bis(trifluoromethane)sulfonimide (TFSI) [59, 76]. Such enhancement could be attributed to the transition from trion-dominated recombination to excitons, and it can also be realized simply by electrostatic doping [61]. It is suggested that defects have no detrimental effect on the PL QY of monolayer TMDCs and all neutral excitons radiatively recombine even in the presence of native defects. This work provides a new pathway for realizing high-performance optoelectronics based on 2D materials. However, PL enhancement has not been realized in selenide-based TMDCs by chemical or electrostatic doping. It is probably due to the presence of different types of defects. It is worth noting that the enhancement of MoSe_2_'s PL could be achieved by exposure to hydrohalic acid vapors, such as HCl, HBr, and HI [77]. The PL intensity of MoSe_2_ dramatically increases more than 30 times by HBr treatment. Low-temperature PL shows that defects within the as-grown MoSe_2_ prohibit the intrinsic exciton emission and the dominant PL peak is mostly from trapped exciton states. For the HBr-treated MoSe_2_, the trapped exciton state is greatly suppressed. The scanning TEM characterization confirms that the Se vacancies in HBr-treated samples are relatively less than the as-grown MoSe_2_ monolayers. It is therefore concluded that the enhancement of PL in MoSe_2_ arises from the release of bound excitons on Se vacancies.

### 3.3. Other Methods for Defect Engineering

Apart from plasma and chemical treatments, there are other methods for defect engineering in 2D materials. In this section, we emphasize on defect engineering triggered by ozone exposure, laser illumination, alloying, and substitutional doping during growth and ion implantation.

**Figure 6:**
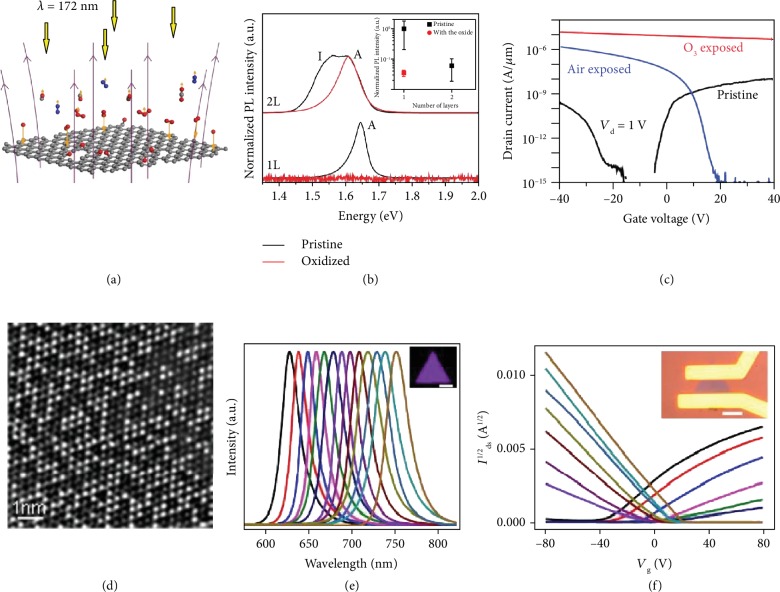
Defect engineering by ozone treatment and alloying. (a) Schematic diagram of ultraviolet ozone treatment. Reproduced from [78]. (b) PL and (c) transfer curves of pristine and ozone-exposed WSe_2_. Reproduced from [79, 80]. (d) STEM image of a typical alloy. Reproduced from [86]. (e) PL and (f) transfer characteristics of WS_2__*x*_Se_2−2__*x*_ alloys. Reproduced from [87].

Ultraviolet ozone treatment has been employed to tailor the optical, electronic, and optoelectronic properties of WSe_2_.  Figure 6(a) shows the schematic illustration of ultraviolet ozone [78]. The bond of oxygen molecules can be broken by ultraviolet light and release two O (^3^P). O (^3^P) is a strong oxidant and can oxidize WSe_2_ with a self-limiting thickness from single to tri-layer. The underlying WSe_2_ remain a perfect hexagonal lattice but hole-doped, resulting in a similar but weaker PL peak as compared to the pristine monolayer [79] (Figure 6(b)). The fabricated FET based on oxidized multilayer WSe_2_ exhibits higher carrier concentration and mobility, owing to the thinning of the barrier width by doping and lower degree of interfacial defects [80] (Figure 6(c)). The photogating effect within WO_*x*_ and underlying WSe_2_ greatly improves the photoresponsivity but with prolonged response time [81]. Oxidation could also be realized via laser illumination. The degree of oxidation can be controlled by laser power. Black phosphorus (BP) flakes with higher oxidation degree show blue-shift of light absorption, indicating the increase of the bandgap and formation of phosphorene oxides [82]. In addition, with the assistance of optical microscopy, mask-free micromachining can be realized. Laser can also heal the Se vacancies in WSe_2_ in oxygen atmosphere [83]. As a result, the conductivity of the WSe_2_ monolayer is increased by 400 times and the photocurrent is enhanced by 150 times.

Substitutional doping of 2D materials can also be realized during the growth process. The Er-doped MoS_2_ synthesized by CVD presents PL emission at 800 nm when excited by a 980 nm laser [84]. This phenomenon is attributed to various energy transfer pathways (localized states) induced by Er doping. The doping of Nb in MoS_2_ synthesized by the chemical vapor transport (CVT) method induces a structural transformation from natural 2H stacking to 3R stacking [85]. The monolayer MoS_2_:Nb hence exhibits strong PL from bound excitons at room temperature, which is usually observed at cryogenic temperatures.

Following this strategy, alloys of 2D materials are also achieved by growth methods. The PL emission of alloys can be tuned by compositions. Different compositions of alloys can be easily identified by TEM, as shown in Figure 6(d) [86]. It has been reported that CVD-grown WS_2__*x*_Se_2−2__*x*_ alloys show tunable PL emission from 626.6 nm (nearly pure WS_2_) to 751.9 nm (nearly pure WSe_2_) (Figure 6(e)) [87]. The n, p, or ambipolar transport behavior can also be easily tuned in alloys. The fabricated FET using WS_2__*x*_Se_2−2__*x*_ alloys shows continual shift in the characteristic output. It shifts from p-type behavior in the WSe_2_-rich phase to n-type in the WS_2_-rich phase and keeps a consistent shift of the threshold voltages (Figure 6(f)). Li et al. have demonstrated that isoelectronic substitution of Mo atoms with W atoms in CVD-grown monolayers of Mo_1−__*x*_W_*x*_Se_2_ (0 < *x* < 0.18) could effectively suppress Se vacancy concentration by 50% as compared to pristine MoSe_2_ monolayers [88]. It reduces defect-mediated nonradiative recombination, facilitates 10 times more intense PL, and increases the carrier lifetime by 3 times.

When defects break the lattice structure of the crystal and form small-sized nanocrystallites (NCs), the fundamental momentum conservation requirement (*q*~0) for the Raman process is relaxed. Phonons away from the Brillouin zone center (*Γ*) will be involved in the Raman scattering, which is well-known as the phonon confinement effect. The phonon confinement effect can be well understood by controlling the size of 2D NCs. Shi et al. studied the Raman spectra evolution of MoS_2_, WS_2_, and WSe_2_ with different doses of ion implantation, which provides an approach to quickly probe phonon dispersion trends of 2D materials away from *Γ* [89, 90]. It is also a reference to understand the confinement effect of different modes in various nanomaterials. The phonon confinement effect of anisotropic 2D materials was well studied using BP as a typical example [91]. The RWL model (common model for the phonon confinement effect) [92] is applicable for anisotropic 2D materials if the phonons in the whole two-dimensional Brillouin zone are properly taken into account. High optical anisotropy of MoS_2_ can be achieved by ion implantation, which is revealed by optical contrast and Raman spectroscopy [93].

## 4. Conclusion and Outlook

In summary, we review the recent research progress of defect engineering in 2D materials. The dynamics of defect transformation are displayed by e-beams with the assistance of EM. e-beams are utilized to introduce point defects like vacancies, SW defects and substitutional impurities, and large area defects such as stoichiometric defects and phase transition. Novel structures like nanoribbons, nanochains, and nanowires can also be fabricated by e-beams. We also give a brief correlation between defects and their influence on optical, electronic, and optoelectronic properties of 2D materials. The modulation of optical and electronic properties by plasma treatment is introduced. It could enhance the PL QY, lower contact resistance, and improve carrier mobility. The healing and doping effects from chemical treatments are also described. Finally, novel properties induced by ozone treatment, laser irradiation, alloying, and substitutional doping during growth are summarized.

Although great progress has been achieved in the last decade, there are still challenges that demand further study. Firstly, despite atomic-scale defects having been successful characterized by TEM and STM, it is still ambiguous to associate the atomic defects with the optical and electronic properties of 2D materials. This might be realized by obtaining different types of defects with uniform distributions. The investigation of an ultrafast carrier dynamic in 2D materials in the presence of defects would be very helpful, e.g., the excitation, relaxation, trapping, recombination, and transport processes, which is crucial for developing high-performance electronic and optoelectronic devices. Finally, the development of large-scale, uniform, and CMOS-compatible approaches for defect engineering is important for the application of 2D materials in electronics and optoelectronics.

## Figures and Tables

**Table 1:** Theoretically calculated knock-on threshold energy for typical 2D materials ^[^23^]^.

Materials	Graphene	*h*-BN		MoS_2_		MoSe_2_	WS_2_
		B	N	S	Mo	Se	S
*E* ^*th*^ (keV)	86	74	84	83	430	175	86
